# Topographic anatomy and intraoperative USG-guided foreign bodies extraction of neglected Molotov cocktail victim: A rare case report

**DOI:** 10.1016/j.ijscr.2023.109009

**Published:** 2023-11-10

**Authors:** Muhammad Ade Junaidi, Fajar Defian Putra, Marcel Prasetyo, Aryo Winartomo, Chintya Mutiara Sari

**Affiliations:** a Trauma Division, Department of Orthopaedics and Traumatology, Faculty of Medicine, Universitas Indonesia, Cipto Mangunkusumo Hospital, Jakarta, Indonesia; b Department of Orthopaedics and Traumatology, Faculty of Medicine, Universitas Indonesia, Cipto Mangunkusumo Hospital, Jakarta, Indonesia; c Musculoskeletal Radiology Division, Department of Radiology, Faculty of Medicine, Universitas Indonesia, Cipto Mangunkusumo Hospital, Jakarta, Indonesia

**Keywords:** Foreign bodies, Molotov cocktail, USG-guided extraction, Topographic anatomy

## Abstract

**Introduction and significance:**

Foreign body implantation resulting from explosive devices is an extraordinary occurrence that often leads to substantial morbidity among the affected individuals. Explosions caused by such devices generate a rapidly propagating blast wave emanating from the point of detonation. This study aims to present a case involving a patient who experienced multiple foreign body implantations as a consequence of a bomb explosion.

**Case presentation:**

A 41-year-old male presented with a history of multiple foreign bodies retained within his body for the past 22 years, originating from a homemade explosive device. At present, he reports weakness in his lower extremities, numbness extending from the umbilical region down to the lower extremities, and fecal incontinence. The patient underwent a surgical procedure for the removal of these foreign bodies, guided by ultrasonography (USG), which lasted for a duration of 12 h.

**Clinical discussion:**

The presence of foreign bodies within the human body incites an inflammatory response. In preparation for surgery, topographic anatomy is delineated through the use of pre-operative CT scans to ascertain the precise locations of these foreign bodies. Subsequently, the removal of these foreign bodies is executed under the guidance of ultrasound.

**Conclusion:**

The extraction of multiple foreign bodies from a patient's body is an infrequent surgical procedure. Meticulous surgical planning, aided by the utilization of X-rays and CT scans for topographic anatomical mapping, is imperative. Employing real-time ultrasound guidance during the procedure serves to minimize blood loss and mitigate potential damage to adjacent structures, thereby enhancing patient safety and reducing the likelihood of surgical complications.

## Highlights

• Foreign bodies implanted into the human body trigger an inflammatory process by recruiting neutrophils, monocyte, and macrophage to the injured area
• Surgery should be carefully planned by topographic anatomy preparation using x-ray and CT scan and compared with USG.
• Multiple foreign body removal is an uncommon procedure to be performed.
• It should be noted that surgical exploration can make large incision and increase the risk of iatrogenic lesion and complications.
• By using the real-time ultrasound-guided procedure and adequate pre-operative planning, it can minimize the amount of bleeding, avoid injury to structures surrounding the foreign bodies and improve patient safety and minimize surgical complications.

## 1 Introduction

The implantation of foreign bodies resulting from a bomb explosion constitutes an extraordinary event associated with a high morbidity rate among its victims. Explosions from bombs generate a rapidly propagating blast wave emanating from the source of the detonation. It is these characteristics of explosions that give rise to various types of injuries observed in the aftermath of such incidents [1,2]. Since this event is inherently complex, the management of foreign body removal from the subject's body poses significant challenges.

Preoperative preparation assumes paramount importance in formulating a comprehensive approach and strategy for intraoperative actions. Such preparation can encompass both imageless and image-based systems. The outcomes of this preoperative preparation are subsequently implemented during the surgical procedure, specifically in the context of ultrasonography-guided foreign body removal.

The primary objective of this study is to present a case involving a patient who had multiple foreign bodies implanted due to a bomb explosion. Additionally, this study seeks to assess the outcomes achieved through preoperative planning employing topographic anatomy in conjunction with ultrasound-guided foreign body removal. It is noteworthy that this case report adheres to the SCARE Criteria for reporting surgical cases [3].

## 2 Case presentation

**Fig. 1:**
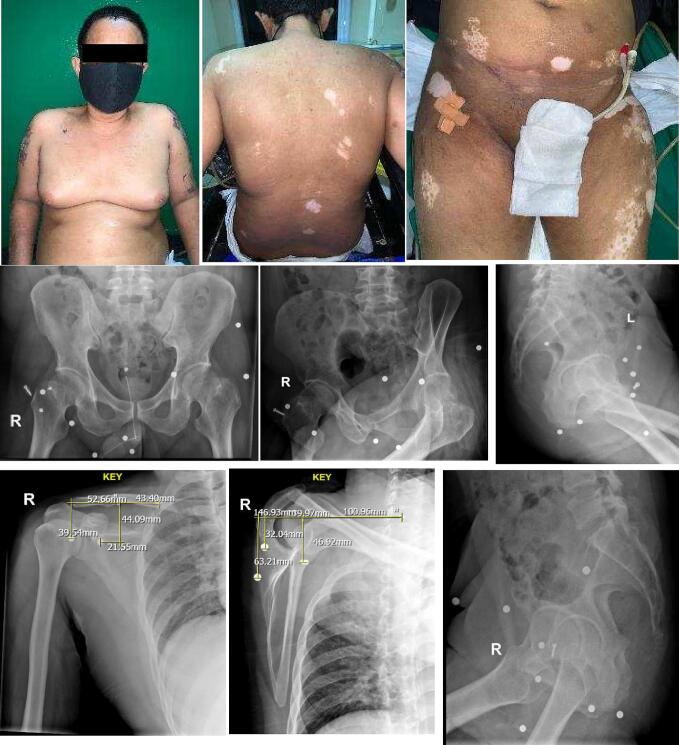
Clinical and radiological picture of male 41 years old with foreign body on right shoulder and hip region. Multiple foreign bodies were easily identified.

A 41-year-old male presented to our hospital with complaints of lower extremity weakness, accompanied by numbness extending from the umbilical region down to the lower extremities, as well as fecal incontinence. According to the patient, these complaints started two weeks after he was involved in a motor vehicle accident, initially manifesting as difficulty in lifting his legs, accompanied by tingling sensations and pain in the left leg. Over the course of two months, the symptoms further evolved to include pain during urination, eventually leading to a two-week period of urinary retention (Fig. 1).

**Fig. 2:**
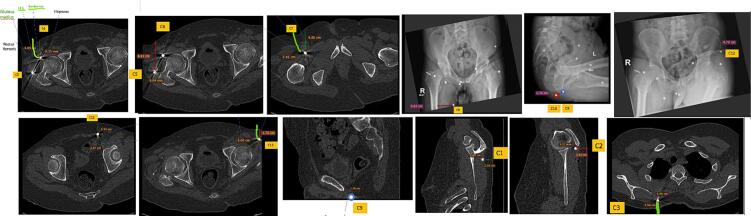
CT scan topographic anatomy of foreign bodies inside the patient.

The patient was referred to our department due to the accidental discovery of multiple metallic foreign bodies within the hip and shoulder regions during an MRI planning session conducted by a neurologist, who initially suspected tuberculosis spondylitis as the underlying cause in this patient. After a more thorough history taking, apparently, the patient had experienced a bomb blast incident 22 years prior, which, inexplicably, had not elicited any complaints at that time. However, given the persistence of neurological deficits and the absence of a definitive diagnosis, it was deemed imperative to subject the patient to a spinal MRI examination. Recognizing that the presence of metallic foreign bodies would hinder this diagnostic process, as well as the marked increase of inflammatory markers of leukocyte count, CRP, ESR, and procalcitonin, a decision was made to proceed with their removal (Fig. 2).

**Fig. 3:**
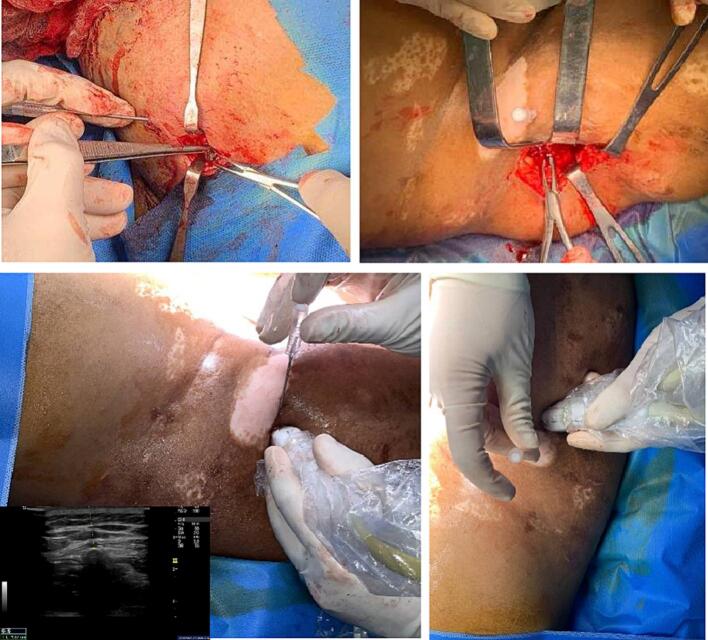
Intraoperative figures of patient undergoing USG-guided foreign body removal.

The patient underwent foreign body removal in August 2022. The foreign objects were extracted through the insertion of an 18G needle into the region, with simultaneous ultrasound (USG) guidance, positioned at a 45-degree angle from the skin. The needle was meticulously maneuvered towards the hyperechoic areas identified on the USG. Following confirmation of the presence of the foreign body, the distance from the skin to the target site was calculated. Subsequently, an incision was made, and a total of 12 small foreign bodies were carefully extracted. Intraoperative bleeding was estimated to be 325 cc. Given the intricate process on the anatomy of the case, the surgical team was composed of vascular, digestive, and urology surgeons, and the procedure endured for a duration of 12 h. Due to the extended surgical intervention, the decision was made to admit the patient to the Intensive Care Unit (ICU) for more intensive postoperative monitoring (Fig. 3).

## 3 Discussion

Cases of multiple foreign bodies due to bomb explosions are rare occurrences. Foreign bodies implanted into the human body trigger an inflammatory process, which can take weeks to months after implantation, depending on the foreign body material. Initially, there is a process of recruiting neutrophils to the injured area triggered by tissue damage and blood extravasation. This process is followed by the recruitment of monocytes, which differentiate into macrophages. A few days later, the macrophages aggregate into giant cells. Several weeks post-implantation, a fibrotic encapsulation by giant cells with M2 macrophages is formed [4].

Since there are multiple foreign bodies in the patient's body, preoperative preparations are made to remove them. Topographic anatomy surgery planning uses optical markers placed at anatomical landmarks on the body [5]. In this method, a previously obtained CT scan is used to determine the location of these foreign bodies. In this case, preoperative planning was carried out using the preoperative CT scan. The distance of each foreign body was measured from the anatomical landmarks and recorded as an intraoperative benchmark [6].

During the topographic anatomy examination, several precautions for performing surgery were noted in Table 1. The first and second foreign bodies were located near the femoral head. Therefore, it's crucial to consider important structures like the femoral nerve and superior gluteal nerve when making incisions and removing foreign bodies. The third and fourth foreign bodies were in the right femoral shaft, and it's essential to take notice of the deep thigh vein, artery branches of the femoral nerve, femoral artery, and vein. The fifth foreign body was located 5.8 cm craniomedial to the right femoral shaft, where the great saphenous vein and obturator nerve are important structures not to be damaged. The sixth and seventh foreign bodies were located anterior-posterior at the posteroinferior of the right pubic rami, where the pudendal artery passed. The eighth foreign body was located superficially from the tensor fascia lata ligament, and care should be taken not to damage the superior gluteal nerve, as with the first and second foreign bodies. The last foreign body in the hip region was located posteroinferior to the left iliac crest, where the sciatic nerve passed, and it should be considered during removal. Three foreign bodies in the shoulder region were located infero-posterolateral to the right scapular spine, inferolateral to the right acromion, and on the medial margin of the right medial scapula. In this region, the axillary nerve is the main nerve that should be avoided when removing foreign bodies.

**Table 1:** Locations of foreign bodies inside the patient.

No	Location
Hip region	
1	Approximately 0.9 cm anterolateral of right femoral head
2	Approximately 0,8 cm anterior of greater trochanter of right femur
3	Approximately 2,4 cm anteromedial of right femoral shaft
4	Approximately 5,8 cm craniomedial of right femoral shaft
5	Approximately 1,5 cm at posteroinferior of right pubic rami
6	Approximately 1,8 cm posterior from the foreign body No 5
7	Approximately 3,1 cm at lateral of left femoral head
8	Approximately 2,6 cm posteroinferior from left iliac crest
9	Approximately 3,1 cm at anterior of left acetabular roof

Shoulder region	
10	Approximately 1.3 cm in inferoposterolateral from right scapula spine
11	Approximately 0.3 cm in inferolateral right acromion
12	Approximately 1.5 cm in medial margin of right medial scapula at the level of T2

During surgery, the removal of foreign bodies was guided by ultrasound. The results of ultrasound imaging showed no difference between USG-guided foreign body removal and topographic anatomy. These methods should be performed because clinical examination cannot accurately identify foreign bodies due to pain, hematoma, and swelling after injury. X-rays can be used to find foreign bodies as they can display radio-opaque objects like glass, metal, and stone. However, only 15 % of non-radioopaque objects can be displayed in X-rays [6]. Ultrasound is the preferred method for investigating foreign bodies in soft tissues since it has a sensitivity and specificity of 90 % and 96 %, respectively. Foreign bodies are displayed as hyperechoic areas with posterior acoustic shadow. However, in cases of chronic trauma, a hypoechogenic halo may be found, formed by granulomatous inflammatory reaction. Ultrasound is highly dependent on the operator and can only display foreign bodies in superficial locations [6,7].

The common management of foreign bodies is through surgery. However, a distinction should be made between open wounds and wounds with small entry holes. In the case of an open wound lesion, surgical exploration is suggested as it allows for lesion assessment and exploration. In cases of foreign bodies with small entry holes, surgical management is usually avoided due to difficulties in detecting small foreign bodies. However, surgical exploration can be performed when there is documented evidence of an associated lesion, as in this case, where surgical exploration was performed because the foreign bodies were not small, and their locations had been precisely mapped. It should be noted that surgical exploration can result in a large incision and increase the risk of iatrogenic lesions and complications. Therefore, surgeons must carefully plan the surgical procedure and assess its risk-benefit before proceeding.

## 4 Conclusion

The removal of multiple foreign bodies is an uncommon procedure. Surgery should be meticulously planned through topographic anatomy preparation, utilizing X-ray and CT scans. The real-time ultrasound-guided procedure minimizes bleeding, reduces the risk of injury to structures surrounding the foreign bodies, and enhances patient safety while minimizing surgical complications.

## Consent

Written informed consent was obtained from the patient for publication of this case report and accompanying images. A copy of the written consent is available for review by the Editor-in-Chief of this journal on request.

## Provenance and peer review

Not commissioned, externally peer-reviewed.

## Ethical approval

Ethical approval for this study was not provided. We only provide written and signed informed consent from patient for publishing the case report. It is as stated in World Medical Association and the statement of 59th WMA General Assembly, Seoul, Republic of Korea, October 2008, in which it is stated that the case reports do not require any ethical clearance.

## Funding

The authors report no external source of funding during the writing of this article.

## Author contribution

Conceptualization: [MAJ, MP]; Methodology: [FDP, MAJ]; Project Administration: [FDP]; Formal analysis and investigation: [AW, CMS]; Resources:[FDP, MP]; Visualization: [MP, AW, CMS]; Writing-original draft preparation:[FDP, MAJ, AW, CMS]; Writing-review and editing: [FDP, MAJ, AW, CMS]; Supervision: [MAJ, MP].

## Guarantor

MAJ: accepts full responsibility for the work and/or the conduct of the study, has access to the data, and controlled the decision to publish.

## Research registration number

NA.

## Conflict of interest statement

We declare that all authors have no conflict of interest to declare.
